# Tono-Pen XL tonometry during application of a suction ring in rabbits

**DOI:** 10.1186/1471-2415-8-14

**Published:** 2008-07-30

**Authors:** Spyridon K Charisis, Harilaos S Ginis, Georgios A Kounis, Miltiadis K Tsilimbaris

**Affiliations:** 1 University Hospital of Heraklion, Eye Clinic, Heraklion, Crete, Greece; 2 Institute of Vision and Optics, University of Crete, Heraklion, Crete, Greece

## Abstract

**Background:**

The purpose of this study is to evaluate the use of Tono-Pen XL in measuring IOP during the application of a suction ring in rabbit eyes with manometrically controlled IOP.

**Methods:**

Tono-Pen XL was calibrated against direct manometry in 10 rabbit eyes. A suction ring was then applied in 4 rabbit eyes and the IOP was determined manometrically during suction ring application at 350 mmHg vacuum pressure. Finally, in 6 catheterized rabbit eyes the IOP was measured with Tono-Pen XL during suction ring application at suction vacuum from 350 to 650 mmHg, while keeping actual IOP stable at 30 mmHg and 60 mmHg.

**Results:**

Linear regression analysis revealed that the Tono-pen XL was reliable for IOPs between 10 and 70 mmHg (R^2 ^= 0.9855). Direct manometry during suction ring application showed no statistically significant variation of Tono-Pen XL readings when the incanulation manometry intraocular pressure changed from 30 mmHg to 60 mmHg and no statistically significant correlation between suction vacuum and IOP measurements.

**Conclusion:**

Tono-Pen XL measurements are unreliable during the application of a suction ring on living rabbit eyes even when the actual IOP is forced to be within the validated range of Tono-Pen XL measurements. This inaccuracy is probably related to altered corneal and scleral geometry and stress.

## Background

Vacuum rings (also referred to as suction rings) are used to stabilize the globe during microsurgical operations such as refractive surgery. In order to achieve suction, the eye's shape needs to conform to the suction ring's shape and therefore as a result of their application-in the general case-the sclera and the cornea are deformed. This deformation results in increased intraocular pressure and increased corneal stress. In terms of clinical safety, an IOP increase of 43 mmHg during suction is considered safe [1]. Moreover it has been suggested that the duration of this increase should be minimal. [2].

The potential complications related to the IOP increase during suction ring application raise the necessity for accurate IOP measurements during operations performed with the aid of a suction ring. Tono-Pen XL is a handheld, computerized, fast and easy to use tonometer, with a small (1,5 mm) transducer tip. It is equipped with a small head that makes it minimally affected by corneal surface abnormalities and corneal central thickness (CCT) [3,4]. Furthermore, compared to other tonometers, Tono-Pen XL showed the least error in estimation of true pressure in New Zealand white rabbits [5]. The Tono-Pen XL provides accurate IOP measurements in a range from 5 to 80 mmHg [6].

The purpose of this study is to test the accuracy of Tono-Pen XL in measuring IOP during the application of a suction ring in rabbit eyes. Additionally, a calibration of the Tono-Pen for the rabbit eye using direct manometry was obtained.

## Methods

All experiments were performed at the Institute of Vision and Optics (University of Crete) using healthy albino rabbits that weighed between 3 and 4 Kg. Animals were treated in accordance to the ARVO Statement for the Use of Animals in Ophthalmic and Vision Research. Initial examination of all rabbits, including baseline measurements of the IOP, showed no pathological findings. Prior to the experiment, all rabbits were anaesthetized using subcutaneous injection of ketamine (40 mg/kg) and xylazine (7 mg/kg). Central corneal thickness (CCT) of each rabbit was measured before the experiment using a contact ultrasound pachymeter (Sonogage, Sonogage Cleveland, OH).

### Tono-Pen XL calibration

Tono-Pen XL (Medtronic Solan, Jacksonville, Florida) was calibrated against direct manometry in 10 eyes of 10 healthy albino rabbits, under general anaesthesia. A sterile 22-gauge intravenous catheter needle, connected to a saline column and to a differential pressure transducer (0–5 psi, 1-ms response time; model 286–686; RS Components, Ltd., Taipei, Taiwan), was inserted in the anterior chamber through the paracentral cornea. The differential pressure transducer was built in with the electronic amplifier and a 12-bit A/D converter in a box communicating with a computer unit through a data interface (RS-232; RS Components, Ltd), and could perform an IOP measurement every 0,15 sec and record them for further processing [7]. Two drops of proparacaine hydrochloride 0.5% were instilled onto the cornea once systemic anaesthesia was achieved, before catheter insertion. The height of the saline column was adjusted in order to get IOP readings ranging from 10 to 70 mmHg (in steps of 10) in the pressure transducer. At each pressure level the IOP was measured eight times using a Tono-Pen XL tonometer, at the 5% level, as indicated by the TonoPen. The lowest and highest values were disregarded and mean values of the remaining six were calculated.

### IOP during suction ring application

Four healthy albino rabbits were enrolled in this experiment. We applied a suction ring, of the same style and configuration as that used in LASIK scaled down to fit rabbit's eye, on the animal's right eye. The suction ring was connected to the vacuum port of a LASIK microkeratome control unit (SCHWIND Eye-tech-Solutions & VIEW-POINT technology AG; Kleinostheim, Germany). A 22-gauge intravenous catheter needle was placed in the anterior chamber through the paracentral cornea, at the border of the suction ring's opening. The catheter was connected to the pressure transducer. Then the suction vacuum was turned on, at a vacuum pressure of 350 mmHg, for approximately 1 minute, while the differential pressure transducer was continually recording IOP.

### Accuracy of Tono-Pen XL measurement during suction ring application

This experiment was performed on 6 eyes of 6 healthy albino rabbits. The suction ring connected to the adjustable suction pump was placed on rabbit's eye. Proparacaine hydrochloride 0.5% was instilled onto the cornea to achieve topical anaesthesia before the application of the suction ring. Once suction was achieved, the 22-gauge intravenous catheter needle, connected to the saline column and to the pressure transducer, was inserted in the anterior chamber of the eye through the paracentral cornea. The height of the saline column was adjusted in order to get IOP readings of either 30 or 60 mmHg from the pressure transducer. At each manometric IOP level the suction vacuum was adjusted from 350 mmHg to 650 mmHg in steps of 50 mmHg. At each step the IOP was measured eight times with the Tono-Pen XL. The lowest and highest values were disregarded and mean values of the remaining six were calculated. The rationale behind hydrostatic regulation of the pressure at two different levels was that the pressure after suction ring application is not constant but is rather characterized by an initial peak followed by a exponential decay. Hydrostatic regulation ensured that the actual IOP was constant during TONOPEN measurements. The procedure lasted approximately 10 minutes for each eye.

## Results

### Tono-Pen calibration

**Figure 1:**
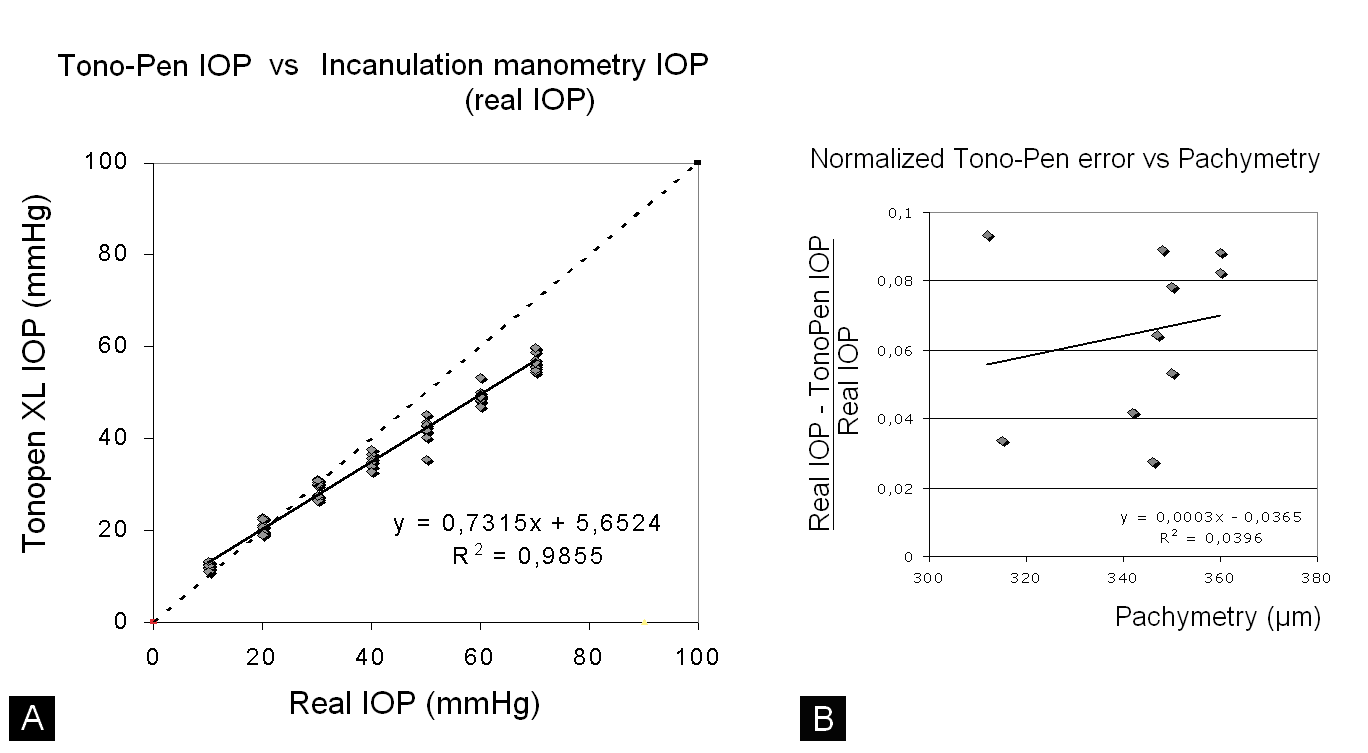
Incanulation manometry IOP versus Tono-Pen XL IOP (A) and Normalized Tono-Pen XL error versus Central Corneal Thickness (CCT) (B).

Linear regression analysis revealed that the Tono-pen XL is reliable for IOPs between 10 and 70 mmHg (R^2 ^= 0.9855). Tono-pen XL underestimates real IOP (Fig. 1A); measured values can be corrected to manometric values using the following formula:

Real_IOP = 1.3472*TONO-PEN-7.0333 [mmHg]. The upper limit of Tono-Pen XL measurements was 90 mmHg.

In order to evaluate the possible contribution of central corneal thickness in Tono-pen XL's offset, we performed a regression analysis between normalized Tono-pen XL error and pachymetry (Fig. 1B). No such correlation could be documented (R^2 ^= 0.03).

### IOP during suction ring application

**Figure 2:**
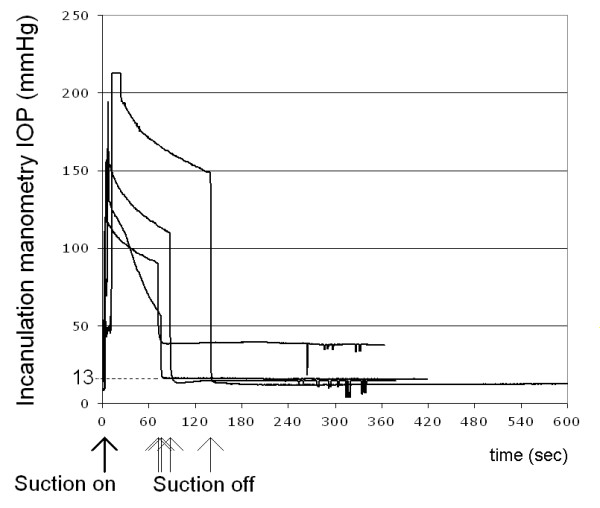
**An IOP spike occurred with vacuum application and peaked immediately.** Peak pressure ranged from approximately 120 mmHg to more than 210 mmHg. After the initial peak, IOP in all eyes demonstrated an exponential decay. After the vacuum was released, IOP returned to normal values.

An IOP spike occurred with vacuum application and peaked immediately. Peak pressure ranged from approximately 120 mmHg to more than 210 mmHg. After the initial peak, IOP in all eyes demonstrated an exponential decay. After the vacuum was released, IOP returned to normal values (Fig. 2).

### Tono-Pen XL during suction ring

As the anterior chamber was connected to the saline solution column, the differential pressure transducer recorded no IOP changes when increasing the suction vacuum from 350 mmHg to 650 mmHg. This finding is reasonable as fluid could either flow into or out of the eye through the catheter depending on the set height of the column and the forces on the ocular wall from the suction ring.

**Figure 3:**
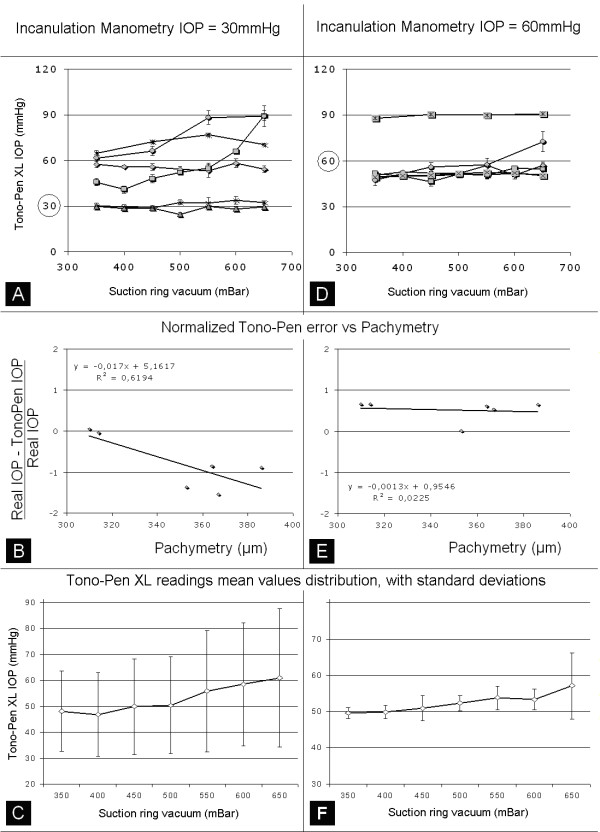
**Tono-Pen XL IOP versus suction ring vacuum pressure when incanulation manometry IOP is stable to 30 mmHg (A) and 60 mmHg (D).** Tono-Pen XL error versus central corneal thickness when incanulation manometry is stable to 30 mmHg (B) and 60 mmHg (E). Tono-Pen XL readings mean values at 30 mmHg (C) and at 60 mmHg (F).

In eyes set at stable IOP 30 mmHg, the Tono-Pen XL recorded a range of values from 26 mmHg to 90 mmHg (Fig. 3A). A regression analysis between normalized Tono-pen XL error and pachymetry shows that the error correlates with the pachymetry at 30 mmHg (Fig. 3B; R^2 ^= 0,6194), but not strongly enough to establish a predictive relationship (Fig. 3B). Although the distribution of mean values of Tono-Pen XL readings seems to correlate with suction vacuum (Fig. 3C), the high values of standard deviations make such a correlation statistically not significant (one-way Anova, P = 0,86 > 0,05).

In eyes set at stable IOP 60 mmHg, the Tono-Pen XL readings are more consistent between 45 and 60 mmHg (Fig. 3D). No correlation between the normalized error (defined as difference between TONOPEN and transducer readings divided by transducer reading) and the pachymetry could be documented at 60mmHg (Fig. 3E; R^2 ^= 0,0225). Again, the distribution of mean values of Tono-Pen XL readings seems to correlate with suction vacuum (Fig. 3F), however one-way Anova showed no statistically significant correlation (P = 0,095 > 0,05).

Finally, for each of the suction vacuum pressures, the independent sample T-test showed no statistically significant variation (P > 0,05) of Tono-Pen XL readings when the incanulation manometry intraocular pressure changed from 30 mmHg to 60 mmHg.

## Discussion

Even if complications related to IOP elevation during suction ring application in refractive surgery are rare, they raise the necessity for more knowledge about IOP changes during such procedures [2,1,8-10]. This knowledge could contribute to increase safety during refractive surgery, not only by influencing patient's inclusion criteria, but also by contributing to the design of new suction rings that work well without excessive increase of IOP.

The rabbit used as an animal model in this study has some known limitations. For example, compared to humans, the rabbit has a different structure to the cornea (no Bowman's) and outflow pathways and high ocular rigidity that could influence IOP behavior during suction ring application. Furthermore, the suction vacuum range used in this study is less than the vacuum settings used in LASIK.

A first step towards understanding IOP behavior during suction ring application would be accurate IOP monitoring. A handheld, accurate and minimally influenced by CCT electronic tonometer such as Tono-Pen XL, theoretically, could be a valid candidate for such IOP measurements [5,6,3]. We calibrated Tono-Pen XL against monometry in 10 animals and we confirmed that the device is accurate for IOP measurements in a range from 10 mmHg to 70 mmHg. These findings are in accordance with the work of others that have shown the accuracy of Tono-Pen XL in this range of measurements [5,6].

The validated range of Tono-Pen XL measurements has an upper limit that is not big enough for IOP measurements during suction ring application. In the current experiment, when the suction ring vacuum was on, the manometricaly measured IOP was characterized by a peak that ranged from approximately 120 mmHg to more than 210 mmHg. IOP during suction ring application has already been measured in human donor eyes [11], in cats [12] and in porcine eyes [13] and was found superior to 90 mmHg. These findings are in accordance to our measurements. IOP levels of this magnitude by far exceed the range of measurements of Tono-Pen XL. In a recent study[14], Acosta et al, evaluated three different tonometers (including the TONOPEN) and concluded that TONOPEN was not accurate or reproducible in estimating IOP in rabbits over the range they tested. However, our findings suggest that an appropriate calibration curve can be obtained, as other investigators have also suggested[15].

Our initial measurements revealed that the pressure levels reached after suction ring application exceeded the measurement range of TONOPEN XL (90 mmHg). It is however possible that this is not the case with other vacuum ring designs. However, besides IOP rise, vacuum ring application results to increased corneal stress. It was hypothesized that even at lower pressure levels the corneal stress associated to the vacuum ring could affect the reliability of TONOPEN XL measurements.

In order to evaluate the tonometer's performance in the conditions that are created during suctioning, we developed an experimental setup that permitted us to set the actual IOP within the validated measurement range of Tono-Pen XL. After suction was achieved in catheterized eyes, we lowered and stabilized IOP to values that can be accurately measured by the Tono-Pen XL. We evaluated whether under these circumstances, the Tono-Pen XL can provide valid IOP measurement. Under these conditions Tono-Pen showed a big variation in measurements. Although there was the impression that Tono-Pen XL becomes more accurate when the real IOP increases, we were not able to document a statistically significant correlation between actual IOP and Tono-Pen XL measurements. This serious limitation of Tono-Pen XL, renders this device unreliable for IOP measurements during suction ring application not only because of its measurement range limits but also because of factors related to the special conditions developed during suctioning of the eye. It is possible that small differences in size and geometry of eyes may result in dramatic differences in the corneal stress distribution during suction ring application. Although a complete analysis of suction ring related corneal stress is a complicated problem, requiring accurate mechanical modelling, it is reasonable to hypothesize that variations in scleral and corneal geometry may result to variations in corneal stress and therefore to variations in the measured IOP with the Tono-Pen XL. It is also probable that the higher the IOP the less scleral and corneal geometry are altered by a suction ring application.

In conclusion, our results show that Tono-Pen XL measurements are unreliable during the application of a suction ring, probably because of altered corneal and scleral geometry and stress.

## Competing interests

The authors declare that they have no competing interests.

## Authors' contributions

SKC has made substantial contributions to data acquisition and has been involved in drafting the manuscript or revising it. HSG has made substantial contributions to conception and design of the study, data acquisition and in revising the manuscript. GAK has made substantial contributions to the statistical analysis and interpretation of the data. MKT contributed to study design, manuscript editing/proofreading.

## Pre-publication history

The pre-publication history for this paper can be accessed here:


